# Signatures of Pancreatic Ductal Adenocarcinoma Uncovered by Integrative Multi-Omics Analysis

**DOI:** 10.3390/cancers18040687

**Published:** 2026-02-19

**Authors:** Benjamin Miao, Tung-Shing Mamie Lih, Yingwei Hu, Hui Zhang

**Affiliations:** Department of Pathology, Johns Hopkins University, Baltimore, MD 21231, USA; bmiao5@alumni.jh.edu (B.M.); tlih1@jhmi.edu (T.-S.M.L.); yhu39@jhmi.edu (Y.H.)

**Keywords:** pancreatic ductal adenocarcinoma (PDAC), single-cell RNA-seq (scRNA-seq), proteomics, phosphoproteomics, RNA-seq

## Abstract

*Background*—Pancreatic ductal adenocarcinoma (PDAC) is among the deadliest malignancies, with a dismal 5-year survival rate. Despite continuous efforts to study its molecular signatures, the high degree of tumor-associated cellular heterogeneity in PDAC introduces extraneous microenvironmental components that complicate analysis. In recent years, multi-omics approaches have shown promise in deconvoluting cellular composition and enabling more specific, comprehensive cancer profiling. *Method*—To better characterize PDAC, we analyzed transcriptomic and proteomic data from 140 tumor tissues with 67 paired normal adjacent tissues and single-cell RNA sequencing data from 73 tumor tissues. *Results*—Using this approach, we successfully attributed molecular signatures to distinct cell-type populations. Overall, we found 59 tumor-cell-derived PDAC molecular signatures and evaluated them for functional relevance, prognostic value, and potential therapeutic implications. Among these, we identified molecular features associated with increased tumorigenic activity and immunosuppression. Moreover, survival analysis of protein phosphorylation and overall expression informed prognostic significance for potential therapeutic targets. Notably, we found that several phosphorylation changes correlate with poor patient survival, suggesting potential paths for therapeutic intervention by targeting protein post-translational modifications. *Conclusion*—Our study provides a detailed understanding of PDAC by characterizing key tumor-specific signatures that could serve as potential targets to improve clinical outcomes for this disease.

## Simple Summary

Pancreatic ductal adenocarcinoma (PDAC), a deadly malignancy, remains poorly characterized. Furthermore, pancreatic ductal adenocarcinoma is often characterized by complex, diverse cell populations that can cause convoluted analysis results. The aim of our study was to use and combine multiple large omics to better understand the molecular feature landscape. Moreover, we specifically identified and characterized 59 signatures from tumor cell populations for pathway functionality as well as prognostics. We ultimately identified three proteins (TRIM29, MUC13, and KRT6A) with significant targetable protein phosphorylation modifications that have notable prognostic implications. Our results provide new perspectives on PDAC functionality and propose new therapeutic avenues.

## 1. Introduction

Pancreatic ductal adenocarcinoma (PDAC) is a severe disease with a poor 5-year survival rate in the United States due to consequential factors such as late diagnosis, potent tumor state, and treatment resistance [1,2,3,4,5]. The current standard of care for PDAC includes chemotherapy regimens such as FOLFIRINOX (fluorouracil, irinotecan, leucovorin, oxaliplatin) or gemcitabine/nab-paclitaxel, and surgical resection when feasible [6]. Treatment of PDAC is often complicated by its characteristic intratumoral cellular heterogeneity, which contributes to treatment resistance [5,7]. As a result, there is a present need to better characterize the PDAC tumor for novel, improved therapeutic intervention.

Approaches involving RNA sequencing (RNA-seq) and proteomics broadly characterize PDAC features at the transcript and protein levels, respectively [5,8]. Further, understanding post-translational modifications (PTMs) can provide key information on regulatory signaling pathways and therapeutically targetable biomarkers, which can be leveraged through phosphoproteomics [8,9]. Nevertheless, a complex system of cell populations convolutes sequencing feature search results, as these technologies are limited to the average expression of the whole sample, lacking in-depth individual cellular information [5].

Single-cell technologies can facilitate deconvolution of cellular identities in the tumor environment, allowing identification of cell-type-specific signatures, such as tumor-cell-specific signatures [5,10,11]. In particular, the addition of single-cell RNA-sequencing (scRNA-seq) information aids in the understanding of cellular composition [12]. ScRNA-seq enables deeper profiling of tumor states with the important ability to distinguish the sample based on cell type, allowing for greater insights into the development of targeted therapies specific to distinct cellular populations [12].

Through multiple omics analyses of cellularity and expression profiles in PDAC at the single-cell level, we hypothesize that identified tumor-cell-specific molecular signatures could reveal new insights into the functional characteristics of the PDAC tumor-specific disease state and provide potential therapeutic targets to improve clinical outcomes. In this study, we performed a comprehensive, exploratory multi-omics analysis, combining RNA-seq and proteomics with scRNA-seq data to compare the PDAC tumor state with normal adjacent tissue (NAT). We characterized features specific to PDAC tumor cells for functional and prognostic purposes. Furthermore, leveraging phosphoproteomics, we identified targetable post-translational modifications of proteins that are correlated with poorer patient outcomes, generating potential avenues for further functional investigation.

## 2. Materials and Methods

### 2.1. Tissues

The analyzed data were obtained from two separate studies by Zhou et al. and Cao et al. [8,11]. The bulk data were obtained from 140 cases of patients with surgically resected and pathology-verified pancreatic cancer (135 PDACs and five pancreatic adenosquamous carcinomas). Sc-RNA-seq data were obtained from primary pancreatic adenocarcinoma samples collected during surgical resection and verified by standard pathology in accordance with the Washington University Institutional Review Board (IRB) at the Washington University School of Medicine in St. Louis (St. Louis, MO, USA). From large-scale omics, tissue-level data outputs were leveraged for downstream analyses.

### 2.2. RNA-seq and Proteomics Data Processing

Differential gene expression analysis comparing tumor and normal adjacent tissue samples was performed with DESeq2 v 1.49.0 [13] from count files obtained from Cao et al. An initial filtering step removed genes with low counts, selecting features with a count intensity ≥ 400. After differential expression analysis, the results data frame was subsequently filtered for feature regulation significance (*p*-value ≤ 0.05, *q*-value ≤ 0.01, and log2FC ≥ 1 or −1 ≤ log2FC). To coalesce the data, we aggregated the multi-omics data to identify conserved differentially expressed features. Upon identification of significantly expressed common features, we performed downstream analyses to characterize the identified features. All analyses was performed via R v4.4.2: https://cran.r-project.org/bin/macosx/.

### 2.3. scRNA-seq Data Processing

From the histologically verified PDAC treatment-naive population data, a Seurat object was created for each sample using Seurat v5.1.0, which was used for subsequent analyses [14]. First, the samples were processed for initial baseline quality via filtering cutoffs for the minimum number of genes expressed (≥200), the minimum number of transcript counts (≥300), the minimum number of expressing cells per gene (≥3), and mitochondrial gene expression over total transcript count (≤10%). All samples were coalesced into a single Seurat object. The merged object was then normalized and scaled. To resolve batch effect, the data were integrated via canonical correlation analysis (CCA) through identification of specific integration anchors based on variable features found through the FindVariableFeatures function (features = 2000 genes). The integrated Seurat was scaled and then reduced to the top 30 dimensions through principal component analysis (PCA), preserving variance with 0.8 resolution. For final clustering, the data were processed via Uniform Manifold Approximation and Projection (UMAP) reduction, then clustered using the FindNeighbors and FindClusters functions. The resulting CCA embeddings were used for downstream analyses. All analyses was performed via R v4.4.2: https://cran.r-project.org/bin/macosx/.

### 2.4. Phosphoproteomics Data Processing

Differential gene expression analysis was performed with DESeq2 v1.49.0 [13] from raw count files for both gene- and site-level phosphoproteomics obtained from Cao et al. The results data frame was used for subsequent analysis to identify phosphorylation of previously identified tumor cell signatures. All analyses was performed via R v4.4.2: https://cran.r-project.org/bin/macosx/.

### 2.5. scRNA-seq Cell Type Annotations

For consistency, cell type assignments were performed by a single person. Key cluster-specific features were identified via the FindMarkers function, identifying only positively associated features with a minimum percentage of 0.5, a minimum number of 5 expressing cells per gene, and a log2FC threshold of 1. Labelling of cell type clusters was done through manual review of the generated set of feature genes in reference to the PanglaoDB database [15]. Identified features were attributed to cell type clusters according to the highest percentage of expressions per cluster. All analyses was performed via R v4.4.2: https://cran.r-project.org/bin/macosx/.

### 2.6. Kaplan–Meier Survival Analysis

Associated clinical data for bulk omics were obtained from Cao et al. Patient data were processed by filtering for patients who were not lost to follow-up and had the official documented cause of death as PDAC. Patient data were stratified by average expression of signature proteins or protein post-translational modifications. Statistical analysis for testing the association between continuous variables and survival outcomes was carried out using the R packages survival v3.7 and survminer v0.4.9 [16,17]. All analyses was performed via R v4.4.2: https://cran.r-project.org/bin/macosx/.

### 2.7. Pathway Analysis

For pathway analysis, we leveraged the R package fgsea v1.34, which uses Gene Ontology biological processes pathways, and generated log2FC statistics of filtered significant signatures from DESeq2 [13,18]. We utilized expression data from both proteomics and scRNA-seq. Significant pathways (*p*-value ≤ 0.05) were obtained based on the expression patterns of tumor cell signatures. Differential expression analysis significant pathway implication (*p*-value ≤ 0.01) results was also evaluated, comparing tumor cell populations with high versus low enrichment of a select population of identified features. Features were evaluated for involvement in select tumor-related pathways and feature-specific top enriched pathways. Analogous pathways were filtered out to allow for the most robust results. All analyses was performed via R v4.4.2: https://cran.r-project.org/bin/macosx/.

## 3. Results

### 3.1. Study Cohort Overview and Multi-Omics Data Workflow

In this study, we used three initial omics datasets: RNA-seq, proteomics, and scRNA-seq [8,11]. The RNAseq and proteomics data were obtained from 140 treatment-naïve tumor tissues, of which 67 were paired with NATs [8]. The single-cell data were derived from a cohort of 25 tumor samples from 7 treatment-naïve patients. Each tumor was sampled two–four times, with each sample being utilized to generate multiple omics [11].

**Figure 1:**
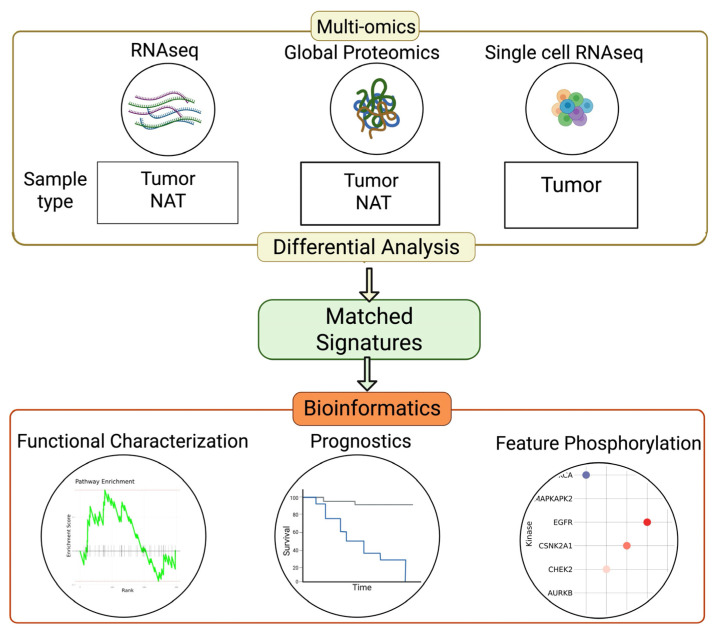
Multi-omics analysis workflow, with study cohort and biological information summary.

To compare the tumor and NAT conditions in pancreatic cancer, we filtered the data for higher purity PDAC tumors using previously established KRAS variant allele fraction (VAF) benchmark cutoffs of 0.075 [8]. Analyses of omics from PDAC and NAT samples identified enrichment of transcripts and proteins (Figure 1, Supplementary Tables S1–1 and S1–2). From scRNA-seq data based on expression profiles of treatment-naïve cases only, we clustered and identified 52,579 cells into 17 distinct cell types, including cancer cells labeled as PDAC (Figure 1, Supplementary Figure S1A, and Supplementary Table S1–3). Downstream analysis involved characterization of signatures with respect to related pathway functionality, prognostic implications, and phosphorylation patterns via phosphoproteomics.

### 3.2. Multi-Omics Integration Reveals Significant, Conserved Differentially Regulated Features Associated with PDAC Tumor Cells and Stromal Features

**Figure 2:**
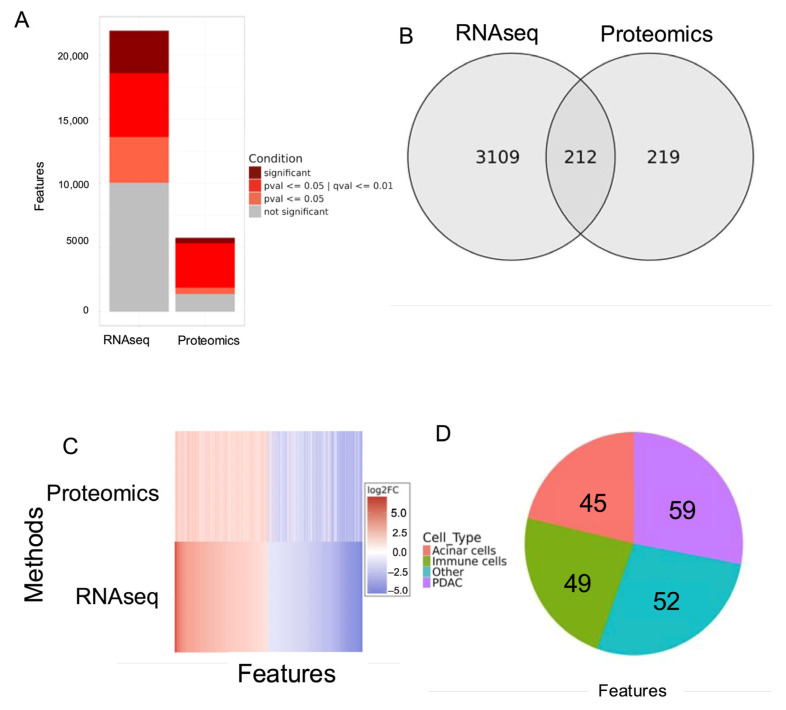
Multi-omics analysis reveals common significantly regulated features with different cell types in pancreatic tumors. (**A**) Gradient bar plot with categories defined by levels of statistical stringency. Significant signatures are defined as *p*-value ≤ 0.05, *q*-value ≤ 0.01, and log2FC ≥ 1 or −1 ≤ log2FC. Not significant signatures fall beyond all set cutoffs. (**B**) Overlapping significantly regulated signatures between omics RNA-seq and proteomics were identified, of which there were 212. (**C**) Significant features ranked by descending mRNA level log2FC were paired to corresponding protein level log2FC to demonstrate a largely conserved expression pattern across two biological levels. (**D**) Cellular identities of 205 features were identified, of which 59 features belong to PDAC tumor cell identities.

Using transcriptomics and proteomics, we performed differential expression analysis using DESeq2 to gain insight into specific regulatory differences between the PDAC tumor disease state and NAT [13]. We filtered the obtained differential features based on imposed statistical cutoffs (*p*-value ≤ 0.05, *q*-value ≤ 0.01, log2 fold change (FC) ≥ 1 or log2FC ≤ −1) to extract significant differential RNA and protein features (Supplementary Tables S2–1 and S2–2). Overall, among the identified features, around 15% of transcripts and 7% of proteins were found to be significant, as shown in Figure 2A. We further filtered these features to identify those significant features shared between RNA and protein levels (Figure 2B). Matching the features by common identity, we obtained a final list of 212 significantly regulated features that are also conserved and shared between transcriptomics and proteomics (Figure 2B). The correlation of target expression between the transcriptomic and proteomic levels demonstrated a largely direct relationship with respect to expression pattern (Figure 2C). This correlation suggests a uniform comparison across the different biological levels for the identified features, as specific regulatory trends are most likely mirrored across either the transcript or protein level.

As part of our study, we were focused specifically on PDAC tumor-cell-attributed features; however, the omics data obtained from bulk tumor samples are often heterogeneous, including many cellular identities [5,19,20]. Thus, to deconvolute the cellularity of the significant signatures obtained from bulk tissue omics, we implemented scRNA-seq data from treatment-naïve patients [11]. Analysis of the scRNA-seq data clustered 17 distinct cell types, including PDAC cancer cells. To characterize cell populations by distinct features, we identified genes with elevated expression, attributing a marker to the cell population with the highest percentage of feature expression (Supplementary Figure S1A). This analysis was applied to all labelled cell types to identify features associated with each cell type (Supplementary Table S2–3). We ultimately were able to attribute 211 out of 212 features (Figure 2B) to a cellular identity (Figure 2D, Supplementary Table S2–3). Shown in Figure 2D, of the 211 bulk signatures attributed to a cellular identity, many were, as expected, associated with tumor microenvironmental cell types, mainly immune cells and acinar cells (Supplementary Table S2–3). Pertaining to our goal, from the scRNA-seq matched features, we also identified 59 PDAC-specific features that are mostly directly correlated in expression trends across transcript and protein levels (Supplementary Tables S2–4 and S2–5 and Supplementary Figure S1B). With these PDAC-associated features, we were able to better characterize specifically the PDAC-associated disease state.

### 3.3. Functional Analyses of Identified Multi-Omics Tumor Cell Signatures Reveal Associated Immune Suppressive Responses and Increase Proliferative Potency

**Figure 3:**
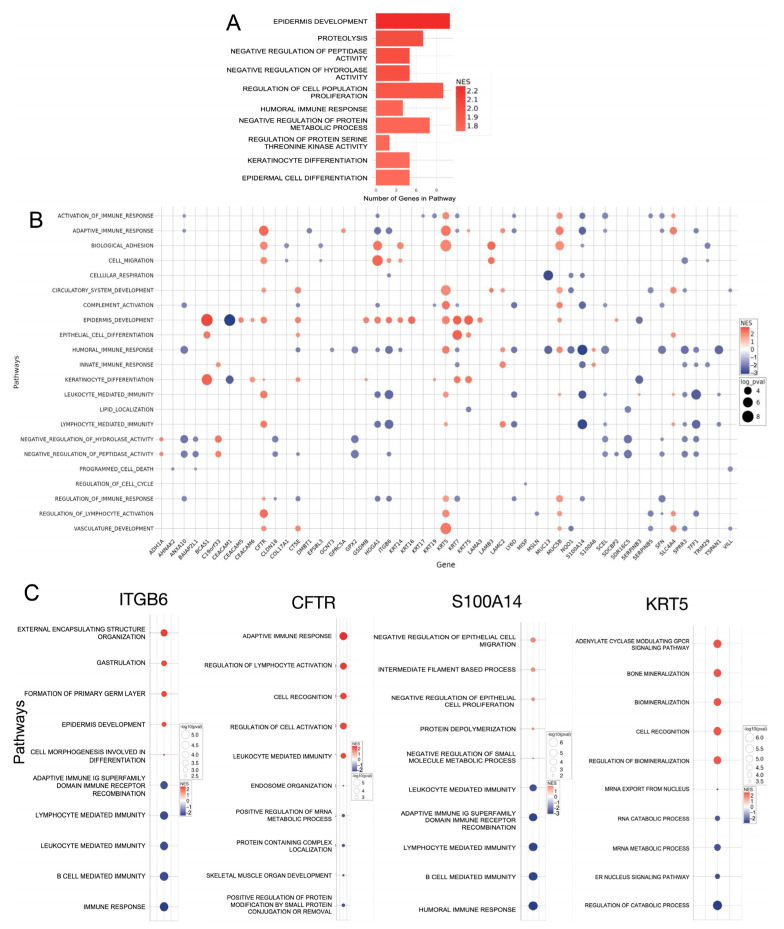
Functional analyses of identified multi-omics PDAC signatures reveal potential associated immunosuppressive responses and treatment resistance. (**A**) fgsea analysis in R provides a more detailed functional characterization of tumor cell signatures. Top pathways were arranged by normalized enrichment score (NES) in descending order. (**B**) PDAC cell signature association with selected tumor-related pathways filtered by *p*-value ≤ 0.01. (**C**) Select signature functionality shown through the top feature-associated pathways, ranked by descending normalized enrichment score, with independently scaled color gradients.

As shown in Supplementary Figure S1C, breaking down the specific identity and expression profiles of signatures at both the transcript and protein levels, we unsurprisingly found many well-established PDAC signatures, such as KRT17/19 and CEACAM5/6, with expected expression patterns in our data [21,22,23]. Interestingly, we also identified several significant features with lesser-known functional associations in PDAC, including KRT5, CFTR, ITGB6, and S100A14 (Supplementary Table S2–4). Thus, we aimed to dissect the functional role of PDAC-associated features. To characterize the specific associated functional pathway implications on the protein level, we implemented fgsea analysis via Gene Ontology biological process pathways [18,24]. Based on the protein expression profiles of the tumor-cell-specific features, we found many of the top listed pathways align with an epithelial-cell-like function with respect to the pathway implications of epidermis development (Figure 3A). Moreover, as shown in Figure 3A, positive enriched pathway functionality regarding epidermis development and cell population proliferation is relevant to the tumorigenic processes, suggesting the active role of several PDAC associated signatures in progressing the disease state. Interestingly, many signatures, such as NQO1 and SERPINB3, which are also associated with poor regulation of enzyme activity (Supplementary Table S2–4), have been shown to correlate with increased resistance to therapeutic intervention [25,26,27].

We sought to further characterize PDAC tumor cells by focusing on the signature-associated functions of all 59 identified features, while mitigating the influence of heterogeneous features. We used scRNA-seq to isolate two specific populations of PDAC cells: one enriched for the feature (log-normalized counts ≥ 1.5) and one negative for the feature. Comparing the differential transcript profiles between these two populations using DESeq2, we then characterized transcriptional expression via pathway analysis using Gene Ontology pathways, filtering for *p*-values ≤ 0.01 to identify tumor-related processes pathway involvement. Overall, the tumor-associated feature functional landscape reveals potential implications in immune suppression via downregulation of specific pathways related to lymphocyte- and leukocyte-mediated immunity (Figure 3B). Furthermore, as shown in Figure 3B, the increased expression of various pathways related to circulatory system and epidermis development portrays increased activity, which can be potentially relevant to increased tumor proliferation.

With respect to specific features, the better-known features we identified had corresponding pathway associations that align with established functionality. For example, CEACAM6 and KRT17 are well-known cancer biomarkers with known roles involving immune evasion and epidermis development, respectively [21,28], which our analysis supports (Figure 3B). Of interest, many signatures that we identified are not well characterized with respect to their functionality in pancreatic cancer. Through pathway analysis, we can elucidate some of the suggested associated functions (Supplementary Table S3). Among the lesser-known signatures, we found particularly significant functional implications for keratin 5 (KRT5), cystic fibrosis transmembrane conductance regulator (CFTR), integrin subunit beta 6 (ITGB6), and S100 calcium-binding protein A14 (S100A14).

ITGB6 is a transmembrane protein with many associations, including involvement in malignant cancer indications including pancreatic cancer [29,30]. With respect to function, ITGB6 has been shown to promote tumor proliferation and invasion, a finding supported by our pathway analysis of both top *ITGB6*-associated pathways and tumor-associated pathways, which show enrichment in proliferative pathways involving development (Figure 3B,C) [29,30]. A closer analysis of pathways identified several notable genes that contribute to pathway enrichment, including *MMP1*, *SMAD3*, and *ITGA2/3* (Supplementary Table S3). *ITGA2/3* are potent signatures that aid in the progression of the tumor disease state [31,32]. Furthermore, *MMP1* and *SMAD3* have important implications for epithelial-to-mesenchymal transition (EMT), which promotes increased tumor migration and invasion [33,34]. As shown in Figure 3C, enrichment in ITGB6 correlates with downregulation of immune response and immune-system-related pathways. Of the implicated immune pathway genes, many are involved in MHC-II antigen presentations, such as *HLA-DPB1*, *HLA-DPA1*, and *HLA-DRA*. Through decreased levels of MHC-II antigen presentation, immune response could be severely attenuated, increasing the severity of the disease state [35].

CFTR is a cAMP-regulated chloride channel that maintains homeostasis of fluids and relevant substrates across the cell membranes [36]. CFTR is most commonly associated with cystic fibrosis; however, established findings suggest CFTR plays an active role in the incidence of various types of cancer, although it is lowly expressed in most tumor tissues [36,37,38]. Our bulk data support these findings in PDAC by demonstrating low expression of CFTR at both the transcript and protein levels (Supplementary Table S2–5, Supplementary Figure S1C). Functionally, CFTR-related mutations and dysfunction have been implicated in many pathways in tumorigenesis, such as metabolism and immune response [38,39]. Here, we investigated the relevance of *CFTR* involvement in tumor-related pathways and the top co-enriched pathways with *CFTR* enrichment. Our results support the potential involvement of *CFTR* in immune regulation, demonstrating that, upon *CFTR* enrichment, several immune response-related pathways are the top-represented functionalities. (Figure 3B,C). The enriched pathway signatures suggest involvement in various adaptive immune functions, from antibody production to cytotoxic responses (Supplementary Table S3). Shown in Figure 3C, as expected, directly paired with *CFTR*-lowered expression are various shifts impacting cellular transport and homeostatic properties [38,40].

S100A14 is a calcium-binding protein that is found to be associated with various cancer indications as well as PDAC [41,42]. In PDAC, S100A14 has been implicated in tumor progression and gemcitabine chemoresistance [43]. However, the underlying mechanisms responsible for the observed functionality of S100A14 remain largely underexplored. On the functional level, we investigated the relevance of *S100A14* in cancer-related pathways as well as top co-enriched pathways with *S100A14* enrichment. Our analysis demonstrates that enrichment of *S100A14* leads to significant downregulation of immune-related processes [41] (Figure 3B,C). Features associated with lowered pathway enrichment are implicated in MHC II antigen presentation, inflammation, and macrophage functionality. Of note, the pathway-related gene analysis also identified downregulated beta-2-microglobulin (*B2M*) as a significant marker relevant to immune pathway activity (Supplementary Table S3). Of the pathway genes for the positively enriched paths, we noted significant enrichment in epiplakin 1 (*EPPK1*) (Supplementary Table S3), suggesting it as a potential key factor influencing pathway function in decreasing tumor-associated proliferative properties [44,45]. Altogether, however, our results suggest that a deficient immune response mechanism is potentially responsible for the *S100A14*-associated tumor progression observed in PDAC [46].

Keratin 5 (KRT5) is an intermediate filament protein responsible for the structural integrity and protection of epithelial cells [47]. KRT5 has been functionally associated with extracellular matrix-aided cell migration in different biological indications, but characterization in PDAC is limited [48,49]. As shown in Figure 3C, *KRT5* enrichment corresponds to enrichment in many proliferative pathways relevant to tumorigenesis, involving vasculature development and epidermis development. Specifically, notable features driving pathway enrichment in our data include *S100A7*, *LOX*, *CDH2*, and *MMP2*, which are potent markers of cell migration and support previously established results [50,51,52,53] (Supplementary Table S3). Among the top enriched pathways associated with *KRT5* enrichment, we identified significant implications in G-protein coupled receptor (GPCR) pathways that have been shown to be responsible for KRT5-positive basal epithelial cell deposition of ECM (Figure 3C) [54]. Furthermore, among the top paths we identified, there was increased potential for calcium signaling, as evidenced by the enrichment of markers relevant to biomineralization, as well as increased mRNA longevity via reduced mRNA metabolism, both of which are integral to the progression of the disease state [55,56,57] (Figure 3C).

Interestingly, we also identified significant implications of immune-related pathways with *KRT5* enrichment (Figure 3B). Among the genes involved in the immune-related pathways, we identified many key genes integral to cell recognition, such as immunoglobulins, which have been implicated as a critical player in tumorigenesis [58,59] (Figure 3C and Supplementary Table S3). We also found a significant co-enrichment of immune-suppressive marker *CTLA4* and immune migratory markers such as *CCR7*, as well as *XCL1*, suggesting an active immunosuppressive state [60,61,62,63,64] (Supplementary Table S3). Ultimately, as indicated by co-enrichment analysis, *KRT5* upregulation may lead to increased immune inactivation and promote the migration of cancer cells.

### 3.4. Prognostic Implications of Tumor-Associated Proteins and Associated Post-Translational Modification Significance

Having identified the functionality of tumor-associated features, we sought to explore their potential clinical significance for prognostics and as drug target candidates. Extending upon our scRNA-seq analysis on the transcriptomic level, we referenced established results from the TCGA Kaplan–Meier survival analysis. Interestingly, of the significantly associated tumor cell signatures, we identified significant prognostic implications for *ITGB6*, *CFTR*, and *S100A14* [28,41,65]. As expected, higher levels of feature expression correlated significantly with poorer survival for patients with PDAC. However, many studies lack full elucidation of protein-level functionality and prognostics. Here we demonstrate that ITGB6, CFTR, and S100A14, on a protein level, lack significant prognostic implication, but, interestingly, we found that KRT5 had significant survival implication on the protein level (Supplementary Figure S2A). Nevertheless, it is important to note that protein results may offer varying results due to potential variation in PTMs that ultimately provide greater functional insights [66,67].

**Figure 4:**
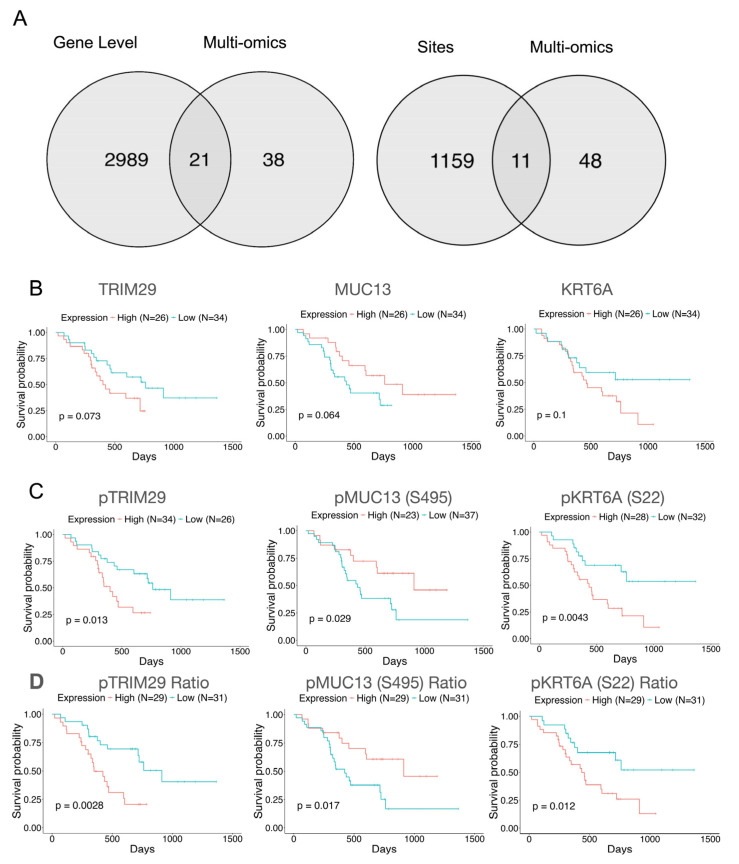
Prognostic implications of tumor-associated proteins and the associated significance of phosphorylated signatures. (**A**) Venn diagram reveals significantly regulated (*p*-value ≤ 0.05, *q*-value ≤ 0.01) phosphorylated tumor cell signatures as well as phosphorylated site level information from the identified phosphoproteome. (**B**) Kaplan–Meier survival curve analysis based on protein expression levels reveals prognostic implications. (**C**) Survival curve analysis based on protein phosphorylation enrichment demonstrates a significant shift in prognostic implication. (**D**) Calculated phosphorylation enrichment over protein expression ratio demonstrates similar significance to protein phosphorylation survival curve analysis.

Thus, to explore PTM-induced shifts in functionality and identify potential targetable markers, we incorporated phosphoproteomics generated from the same bulk tissue omics samples and corresponding signature kinase data into our analysis. First, analyzing the phosphoproteomics data, we sought to identify PDAC-associated genes with significantly altered phosphorylation signatures. Of the 59 PDAC cell-associated transcriptomic and proteomic features (Figure 2D), we found that 21 were also regulated as phosphorylated signatures (Figure 4A and Supplementary Table S4–1). In addition, we identified 11 phosphorylation features regulated at the phosphorylation site level (Figure 4A and Supplementary Table S4–2). To explore the clinical relevance of protein phosphorylation, we implemented Kaplan–Meier survival estimation. Interestingly, we found that 1 of the 21 phosphoproteins and 2 of 11 phosphosites showed prognostic implications both on protein and phosphorylation levels (Figure 4A–C). Furthermore, we calculated the phosphorylation-to-protein expression ratio for the phosphorylated features to ensure that the observed patterns were not attributable to artifacts of protein expression. We ultimately observed a similar pattern, indicating that feature phosphorylation drives the shift in prognostic implication (Figure 4D). Overall, we found that, beyond protein abundance, phosphorylation induction in the identified signatures demonstrates greater significant prognostic stratification.

Keratin 6A (KRT6A) is a type 2 keratin protein that is involved in the proliferation and progression of various cancers, with specific associations with PDAC as well (Supplementary Figure S2B) [68,69,70]. In PDAC, KRT6A has been functionally associated with tumor progression and chemoresistance [70]. Furthermore, gene-level analysis has indicated potential prognostic implications of *KRT6A* [71]. Interestingly, however, our study demonstrates that, on a protein level, the patient population with enriched KRT6A had non-significant poorer prognosis (*p*-value = 0.1) (Figure 4B). Upon induction of phosphorylation of KRT6A at site S22, patient survivability significantly drops, demonstrating the prognostic value of phosphorylated KRT6A (pKRT6A) (Figure 4C,D).

Mucin 13 (MUC13) is a transmembrane glycoprotein that is often expressed in a variety of epithelial carcinomas [72]. Unsurprisingly, on both the transcriptional and protein levels, MUC13 is significantly elevated in tumors relative to NATs (Supplementary Figure S1C, Supplementary Table S2–5) [73]. Regarding the clinical significance of MUC13 shown in Figure 4, intriguingly, high levels of phosphorylation at S495 of MUC13 (pMUC13) showed a better prognosis (Figure 4C,D). With respect to modification functionality, some findings demonstrate that a lack of MUC13 phosphorylation via PKC negatively affects membrane integrity, suggesting worse pathogenesis of the disease state [74].

A tripartite motif containing protein 29 (TRIM29) is responsible for the regulation of the ubiquitylation of short-lived regulatory proteins [75]. Further, TRIM29 is upregulated in various cancers and is a key factor in tumorigenic processes, as our pathway results, which include increased vasculogenesis and epithelial maintenance, also suggest (Supplementary Figure S2B) [76]. Some studies demonstrate the ability of TRIM29 to confer resistance to gemcitabine, a chemotherapeutic agent, in PDAC [75]. Despite specific associations to malignant functional properties with respect to the disease state, our results demonstrate lesser prognostic significance of the protein expression profile (Figure 4B), which aligns with established results showing that unmodified TRIM29 protein, on a prognostic level, yields mixed results [76]. As shown in Figure 4C,D, high TRIM29 phosphorylation (pTRIM29) demonstrated significant prognostic implications. As a potential explanation, the established literature indicates that pTRIM29 confers resistance to ionizing radiation [77], further supporting its clinical importance.

## 4. Discussion

Characterization of PDAC tumors is essential for expanding knowledge of the pathogenesis of PDAC and for providing tools to inform diagnostics, prognostics, and therapeutics [78,79]. However, studies are challenged by many factors, such as cellular heterogeneity, which can result in non-specific molecular signatures [79,80]. In an exploratory analysis, by applying multiple omics and single-cell technologies, we were able to summarize the heterogeneity of the PDAC environment. From the analysis of differentially expressed genes found in bulk omics, we found many features were associated with cell identities other than PDAC cancer cells, confirming the influence of heterogeneous cellular identities.

Using transcriptomics, proteomics, and scRNA-seq, we characterized the expression profile and cellular composition of tumor tissues from PDAC patients. We ultimately identified 59 unique PDAC tumor-cell-derived signatures that were significantly regulated in association with the tumor. The identified features showed significant pathway associations with the progression of the disease state, including epidermal development and immune suppression. Specifically, molecular signatures such as ITGB6, CFTR, S100A14, and KRT5 were most strongly associated with shaping a poor tumor state. We showed that, in *ITGB6*-enriched cell populations, there was high expression of potent EMT proteins. Supporting this indication, we also observed in the related cell differentiation pathway function co-enrichment in laminin isoforms *LAMA3*, *LAMB3*, and *LAMC2* (Supplementary Table S3), which have specific implications in shaping EMT in tumor environments [79,80,81,82,83]. With respect to CFTR, given the lowered expression of CFTR in the tumor environment, our results suggest potentially impaired immune response in the PDAC disease state through lowered adaptive immune response. *S100A14*-associated pathway enrichment demonstrated potential for limiting immune response. Specifically, in enriched pathway-associated genes, we observed key feature enrichment of immune response, such as *B2M* downregulation, which has been associated with absent MHC I antigen presentation, impairing recognition of CD8+ T cells and leading to immune evasion [84]. With respect to pathways directly correlated with *S100A14* enrichment, we identified many pathway functions associated with reduced tumorigenic potential, such as reduced epithelial migration. This result aligns with previous findings of *S100A14*-related functionality in other cancer indications, warranting further verification in the PDAC context [85]. Nevertheless, these results, together, further shed light on the suggested functional role of *S100A14* enrichment in hindering immune response. Lastly, with respect to KRT5, a feature that has not been extensively studied in PDAC, we highlight its key functional involvement in processes such as vasculature development and immune response, which may be implicated in severe disease states. Overall, further research is necessary to elucidate the implications of feature-associated mechanistic shifts in cellular functionality and disease pathogenesis.

In addition to protein-level information, we also implemented phosphoproteomics to examine the significance of survivability for post-translational modifications of the tumor-cell-associated targets. Analyzing site-specific and overall levels of phosphorylation, we found three candidates from our previous list of 59 features (TRIM29, MUC13(S495), and KRT6A(S22)). Leveraging the phosphoproteomics, we identified that, upon phosphorylation of the particular signatures, prognosis significantly worsens. From established results, pTRIM29 has been shown to confer radioresistance in PDAC [77]. TRIM29 phosphorylation under the control of the MAPKAPK2 (MK2) kinase [77], given its biological and prognostic importance, supports the promising potential and relevance of the MK2–pTRIM29 relationship as a therapeutic target for PDAC treatment. The functional implications of phosphorylated MUC13 have not been clearly elucidated in the PDAC environment. With respect to mechanistic regulation, S495 is located in the cytoplasmic domain of the MUC13, which presents a protein kinase C (PKC) phosphorylation motif, suggesting PKC is responsible for the phosphorylation modification, although the subtype is not yet known [74,86]. Despite some suggestions of MUC13 phosphorylation effects on membrane integrity [74], there is a current dearth of knowledge on the resulting functionality upon induction of MUC13 phosphorylation within PDAC. Nevertheless, given the functional importance and prognostic significance of pMUC13, MUC13 is a potentially promising therapeutic target, perhaps warranting further exploration. The mechanistic regulator of pKRT6A phosphorylation at site S22 and the resulting functional shift implications have not been well documented. Given the demonstrated prognostic importance of pKRT6A, pKRT6A could be of interest for further exploration and a potential viable therapeutic target. Overall, our work sheds light on the importance of PTMs by providing support for TRIM29 and MUC13, as well as their respective kinases, MK2 and PKC, as potential therapeutic avenues for PDAC. Future work will be needed to further explore the functional implications and therapeutic values of pTRIM29, pMUC13, and pKRT6A.

## 5. Conclusions

Our study provides a comprehensive analysis of PDAC tumor expression to identify tumor-cell-specific features. These features were analyzed for pathway and prognostic implications to reveal key tumor-related functionality. Furthermore, through survival analysis of signature phosphorylation, we identified TRIM29, MUC13, and KRT6A as potential avenues for therapeutic intervention to improve patient outcomes.

## Data Availability

Original bulk tissue omics and single-cell RNAseq data can be found in https://www.linkedomics.org/#/ and https://data.humantumoratlas.org/, respectively.

## Supplementary Materials

The following supporting information can be downloaded at: https://www.mdpi.com/article/10.3390/cancers18040687/s1, Figure S1: Identification of PDAC-specific transcriptomic and proteomic signatures; Figure S2: PDAC-specific protein and phosphorylation signatures. Table S1: Bulk data omics and annotations; Table S2: Differential analysis results and PDAC-associated data; Table S3: PDAC feature associated pathways; Table S4: Phosphoproteomics data and analysis results.
