# Supplementary material for: Signatures of Pancreatic Ductal Adenocarcinoma Uncovered by Integrative Multi-Omics Analysis
Source: Cancers (Basel). 2026 Feb 19;18(4):687. doi: 10.3390/cancers18040687 (PMC12939947; doi:10.3390/cancers18040687)
Supplement: Supplementary file 1 [file cancers-18-00687-s001.zip › Supplementary figures.pptx]

## Slide 1
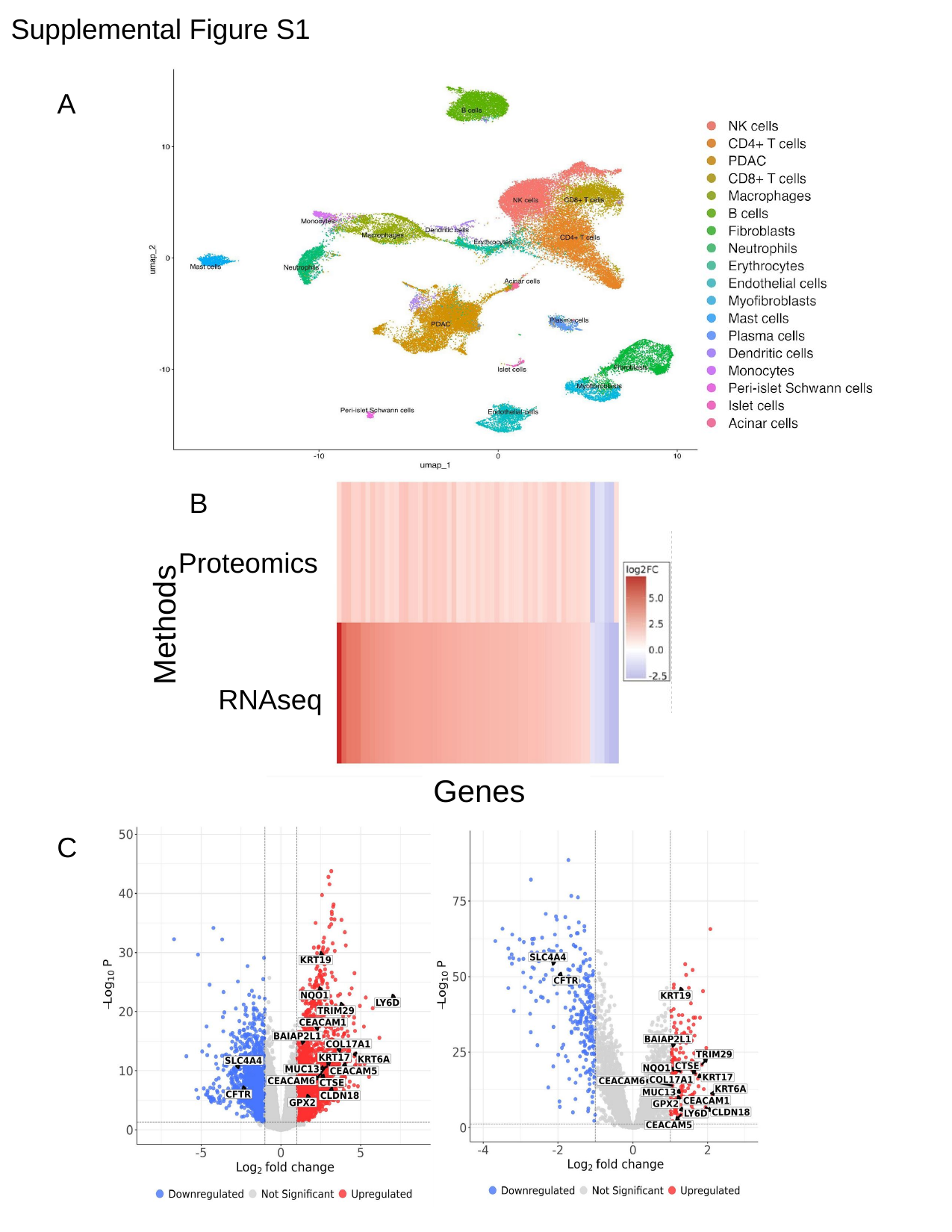

Supplemental Figure S1
A
Proteomics
Methods
RNAseq
Genes
B
C​

## Slide 2
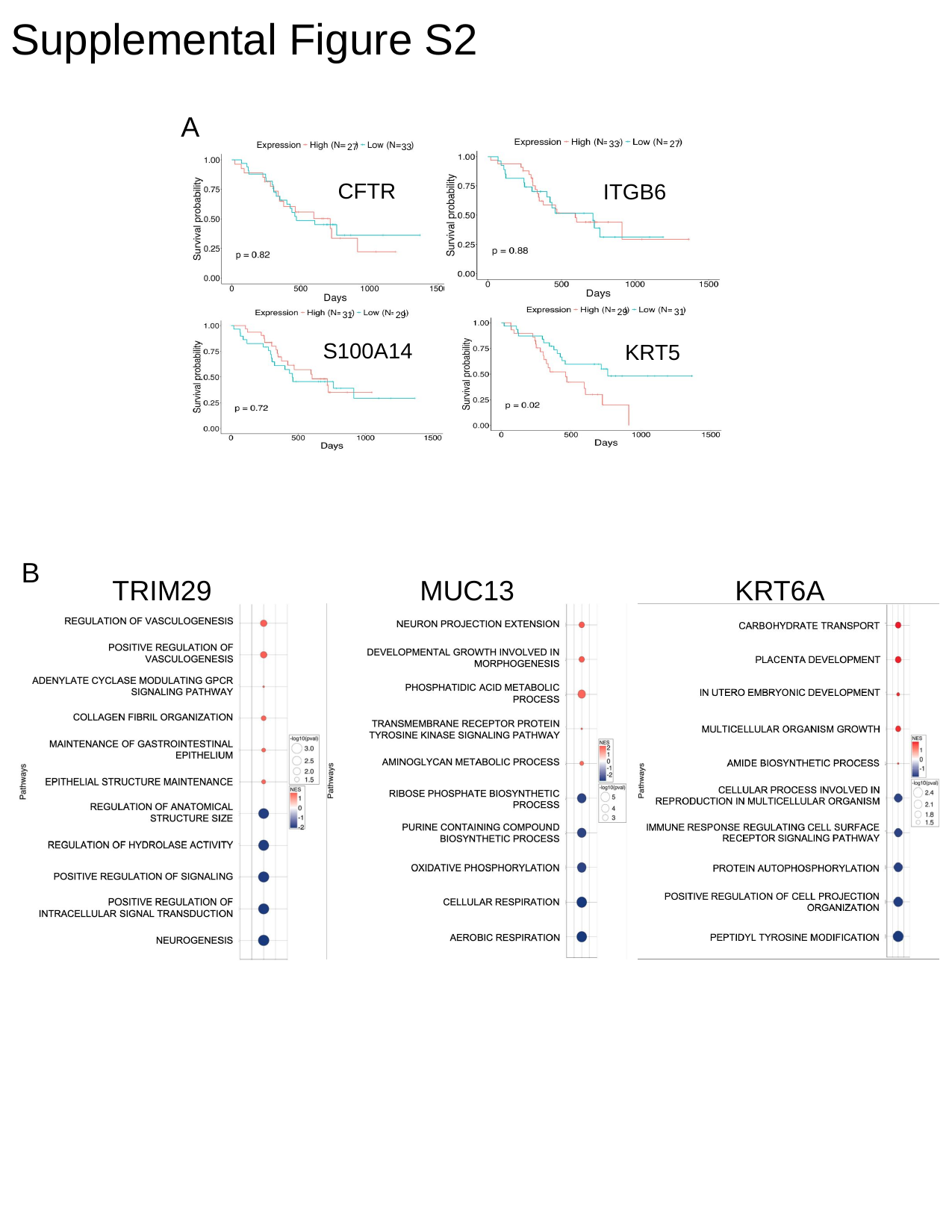

# Supplemental Figure S2
A
33
27
ITGB6
27
33
CFTR
CFTR
29
31
KRT5
31
29
S100A14
B
TRIM29
MUC13
KRT6A
